# Supplementary material for: User abnormal behavior recommendation via multilayer network
Source: PLoS One. 2019 Dec 3;14(12):e0224684. doi: 10.1371/journal.pone.0224684 (PMC6890179; doi:10.1371/journal.pone.0224684)
Supplement: Attachment — Submitted filename: Response to Reviewers.pdf [file pone.0224684.s002.pdf]

Response to Reviewers and Editors' Comments

& List of Changes in the Revised Paper

Title: Privacy-Preserving Recommendation of User Abnormal Behavior Under Multilayer Network

Authors: Chengyun Song, Weiyi Liu, Zhining Liu, Xiaoyang Liu

The authors thank the reviewers and editors for their comments and suggestions. We have revised the paper following the comments and suggestions. Following is our response and also the list of changes that we have made in the revised version. The changes, including newly added parts, are in red font in the revised manuscript.

Review Comments to Author:

Reviewer 1:

1. Reconcile the 15% and 85% satisfaction mentioned in the conclusion

> of 1.0, and an F1-score of around 0.94. Also, we have also reported a linear time complexity for our system. Last, we deploy our system on the "Wenjuanxing" crowd-sourced system and "Amazon Mechanical Turk" for other users to evaluate in all aspects. The result shows that almost all feedbacks have achieved top 15% satisfaction.
>
> Sticky Note 2019/7/26 5:19:52 选项
>
> Reconcile the 15% and 85% satisfaction mentioned in the conclusion
>
> **1 Introduction**
>
> With the recent growth of web activity such as online sh[illegible] becoming more and more necessary to detect abnormal [illegible] example, we can safeguard privacy for real users by dist[illegible]

Response:

Sorry to bring some mistakes and ambiguous here. We meant to say that according to our proposed practical system is highly friendly to uses, as we use "85% satisfaction" to point out there are a large amount of users that do accept our proposed system, and only "15%" of users which are NOT satisfied. To eliminate the ambiguous, we use "up to 85% satisfaction" in the abstract and conclusion

2. Citation missing

> • **Abnormal behavior recommendation**: If there is any abnormal nodes in the current multilayer network, this task recommends them. At the same time, it also shows the group score for devices[4], calculates the security score for the user, and provides a report for current suspicious activities. Here, the security score is defined as $s = 1 - \frac{|abnormal\_nodes|}{|all\_nodes|}$.
>
> Sticky Note 2019/7/26 4:40:59 选项
>
> citation
>
> [4] We group devices together if they share the same score.

Response:

In the paper, we define a simple and way (the percentages of abnormal nodes) to evaluate the security scores in multilayer network. So, there is no citation here.

3. From the decision box " If True". The flow chart did not say anything about 'if false". Marking

the suspicious node is not enough but creating a library of suspicious nodes to be verify for subsequent logins

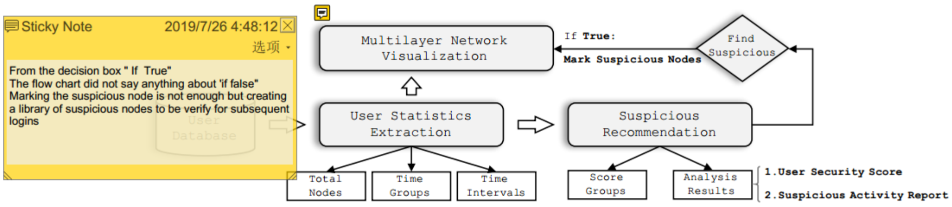

Fig 4. Architecture of visualization system.

Response:

For "If false", the program will ignore the current user. We have updated this figure in the revised paper. And if the system has found a "suspicious" node, it will raise an alert to alarm the user, as the final decision should be made by user himself. The new figure is:

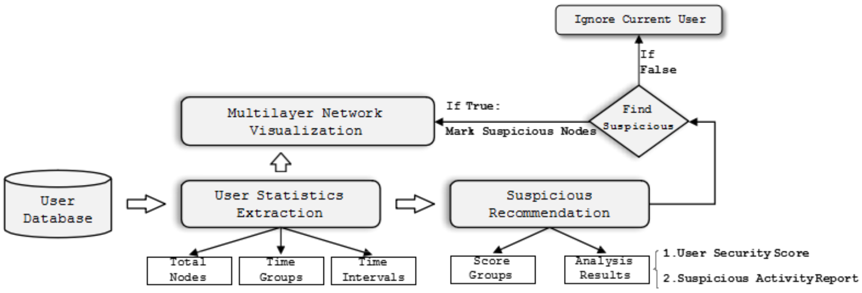

4. Give the scenario of medium level noise in this method

Table 2. Comparison with state-of-the-art algorithms

Related properties	**Proposed method**	Robust deep auto encoder	ML-based algorithms	Asynchronous MTL
1. Privacy-preserving	Embedded	Required	Embedded	Embedded
2. Volume of input features	Few	Large	Large	Large
3. Unsupervised	Yes	Yes	No	No
4. Training-free	Yes	No	No	No
5. Analyzes different sources	Yes	No	[illegible]	Yes
6. Noise-resistance	Medium	High	[illegible]	High

Notes: ML = machine learning, MT[illegible]

Sticky Note 2019/7/26 4:53:22 选项
Give the scenario of medium level noise in this method

4.4 Abnormal Device De[illegible]

Response:

Here we use "medium" as the opposite of "High". In fact, due to the fact that our proposed method (privacy-preserving) and the ML-based algorithms needs all the information to learn the user behavior, these methods have possibilities to absorb noises information as well. Hence, we have replaced term "medium" as "Not Effected".

5. Cite standard that support this assertion

Table 4. System efficiency

	Min T(ms)	Avg T (ms)	Max T (ms)
Step 1 Construction	0.19	5.85	17.51
Step 2 Recommendation	0.61	2.23	4.45

Notes: The overall time to system construction and recommendation steps is around 10ms; from the system operator's point of view, this indicates the proposed system is fast enough for large-scale deployment.

Sticky Note 2019/7/26 5:32:36 选项
Cite standard that support this assertion

Since there is no ground truth per user, we inject synthetic suspicious device logs into normal logs for a user, construct the multilayer network based on these logs, and use precision rate, recall rate, and F1-score [43] to evaluate the performance of the system. Here, for synthetic abnormal devices, the logged features such as encrypted

Response:

We have added the cite paper to support the assertion in the revised version.

6. Reconcile with abstract

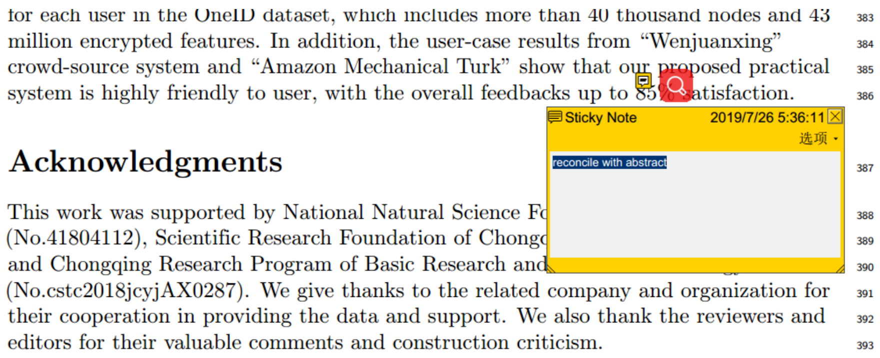

for each user in the OneID dataset, which includes more than 40 thousand nodes and 43 million encrypted features. In addition, the user-case results from "Wenjuanxing" crowd-source system and "Amazon Mechanical Turk" show that our proposed practical system is highly friendly to user, with the overall feedbacks up to 85% satisfaction.

Acknowledgments

This work was supported by National Natural Science Fo[illegible] (No.41804112), Scientific Research Foundation of Chongq[illegible] and Chongqing Research Program of Basic Research and[illegible] (No.cstc2018jcyjAX0287). We give thanks to the related company and organization for their cooperation in providing the data and support. We also thank the reviewers and editors for their valuable comments and construction criticism.

Response:

Sorry to bring ambiguous here. We have used "up to 85% satisfaction" in the abstract which is same to the conclusion.

Reviewer 2:

1. Page 1, line 6-7

   Change "machine learning algorithms" for "machine learning (ML) algorithms"

Response:

I have added the abbreviation in the revision (Page 1 line 6-7).

2. Page 1, line 7

   Change "SVM" for "Support Vector Machine (SVM)"

Response:

I have added the abbreviation of "SVM" in the revision (Page 1 line 7).

3. Page 1, line 9

   Change "machine learning algorithms" for "ML algorithms"

Response:

I have used the abbreviation in the revision (Page 1 line 9).

4. Page 1, line 13

   Change "IP adresses" for "Internet Protocol (IP) addresses"

Response:

I have given the full name of IP in the revision (Page 1 line 14).

5. Page 1, line 15

   Change "machine learning models" for "ML models"

Response:

I have used the abbreviation of "ML" in the revision (Page 2 line 15).

6. Page 2, line 18

   Change "machine learning methods" for "ML methods"

Response:

I have used the abbreviation of "ML" in the revision (Page 12 line 18).

7. Page 2, line 27

   Change "machine learning algorithms" for "ML algorithms"

Response:

I have used the abbreviation of “ML” in the revision (Page 2 line 27).

8. Page 2, line 30

“…over time1.” Confirm in the Plos One standards if footnotes can be used.

Response:

We have not found the standards about the footnotes. However, this standard is used in other journals. So I do no changes and keep the original format in the revision (Page 2 line 30).

9. Page 2, line 39

Change “machine learning” for “ML”

Response:

I have used the abbreviation of “ML” in the revision (Page 2 line 39).

10. Page 2, line 55

“The system is available at https://github.com/StoneSongLucky/Private Preserving Outlier Behavior Detection” The link is unavailable.

Response:

The correct links are

https://github.com/StoneSongLucky/Private_Preserving_Outlier_Behavior_Detection

and

https://github.com/Liu-WeiYi/Private_Preserving_Outlier_Behavior_Detection

The link in the paper is right and available.

11. Page 2, line 56-62

Delete: “The remainder of the paper is organized as follows. In Section 2, we discuss recent work on user abnormal behavior recommendation. Section 3 describes the whole proposed model, and describes network construction (Section 3.3), network analysis (Section 3.4), outlier recommendation (Section 3.5) and a visualization practical system (Section 3.6). In Section 4, we present a thorough evaluation of the proposed privacy-preserving recommendation system. Finally, we conclude and describe future work in Section 5.”

Response:

I have deleted those contents in the revision.

12. Page 3, line 65

Change “machine learning” for “ML”

Response:

I have used the abbreviation of “ML” to replace ‘machine learning’’ in the revision (Page 2 line 57).

13. Page 3, line 71

Change “machine learning (ML) models” for “ML models” - The abbreviation should appear the first time the word is cited in the manuscript (as per the previous changes I am suggesting).

Response:

I have revised those mistakes in the revision (Page 3 line 64).

14. Page 10, line 262

Change “machine learning-based algorithms” for “ML-based algorithms”

Response:

I have used the abbreviation of “ML” in the revision (Page 10 line 254).

15. Page 10, line 266

Change “machine learning-based algorithms” for “ML-based algorithms”

Response:

I have used the abbreviation of “ML” in the revision (Page 10 line 257).

16. Page 11, line 283

Change “machine learning-based algorithms” for “ML-based algorithms”

Response:

I have used the abbreviation of “ML” in the revision (Page 11 line 274).
